# Bayesian Calibration of Simultaneity in Audiovisual Temporal Order Judgments

**DOI:** 10.1371/journal.pone.0040379

**Published:** 2012-07-09

**Authors:** Shinya Yamamoto, Makoto Miyazaki, Takayuki Iwano, Shigeru Kitazawa

**Affiliations:** 1 Human Technology Research Institute, National Institute of Advanced Industrial Science and Technology (AIST), Tsukuba, Japan; 2 Research Institute for Time Studies, Yamaguchi University, Yamaguchi, Japan; 3 Department of Neurophysiology, Juntendo University Graduate School of Medicine, Tokyo, Japan; 4 Dynamic Brain Network Laboratory, Graduate School of Frontier Biosciences, Osaka University, Osaka, Japan; 5 Department of Brain Physiology, Graduate School of Medicine, Osaka University, Osaka, Japan; 6 Center for Information and Neural Networks, Osaka University, Osaka, Japan; Bielefeld University, Germany

## Abstract

After repeated exposures to two successive audiovisual stimuli presented in one frequent order, participants eventually perceive a pair separated by some lag time in the same order as occurring simultaneously (lag adaptation). In contrast, we previously found that perceptual changes occurred in the opposite direction in response to tactile stimuli, conforming to Bayesian integration theory (Bayesian calibration). We further showed, in theory, that the effect of Bayesian calibration cannot be observed when the lag adaptation was fully operational. This led to the hypothesis that Bayesian calibration affects judgments regarding the order of audiovisual stimuli, but that this effect is concealed behind the lag adaptation mechanism. In the present study, we showed that lag adaptation is pitch-insensitive using two sounds at 1046 and 1480 Hz. This enabled us to cancel lag adaptation by associating one pitch with sound-first stimuli and the other with light-first stimuli. When we presented each type of stimulus (high- or low-tone) in a different block, the point of simultaneity shifted to “sound-first” for the pitch associated with sound-first stimuli, and to “light-first” for the pitch associated with light-first stimuli. These results are consistent with lag adaptation. In contrast, when we delivered each type of stimulus in a randomized order, the point of simultaneity shifted to “light-first” for the pitch associated with sound-first stimuli, and to “sound-first” for the pitch associated with light-first stimuli. The results clearly show that Bayesian calibration is pitch-specific and is at work behind pitch-insensitive lag adaptation during temporal order judgment of audiovisual stimuli.

## Introduction

After repeated exposures to a constant lag between auditory and visual signals, the brain recalibrates subjective simultaneity such that the signals separated by the delay are perceived as simultaneous [1], [2], [3], [4], [5], [6], [7], [8]. This so-called lag adaptation appears to be helpful for binding two signals that have arrived in the brain with a certain delay but actually originated from a single event. Such a delay is typically observed between auditory and visual signals originating from a single event at a distance. A difference in conduction time in the peripheral nervous system is another source of the signal delay.

However, lag adaptation was not observed in judging the order of two tactile stimuli, each of which was delivered to either the left or right hand. Rather, participants showed completely opposite perceptual changes that conformed to a Bayesian integration theory in that two simultaneous stimuli were perceived as having occurred in the order of the most frequent lag [9].

**Figure 1:**
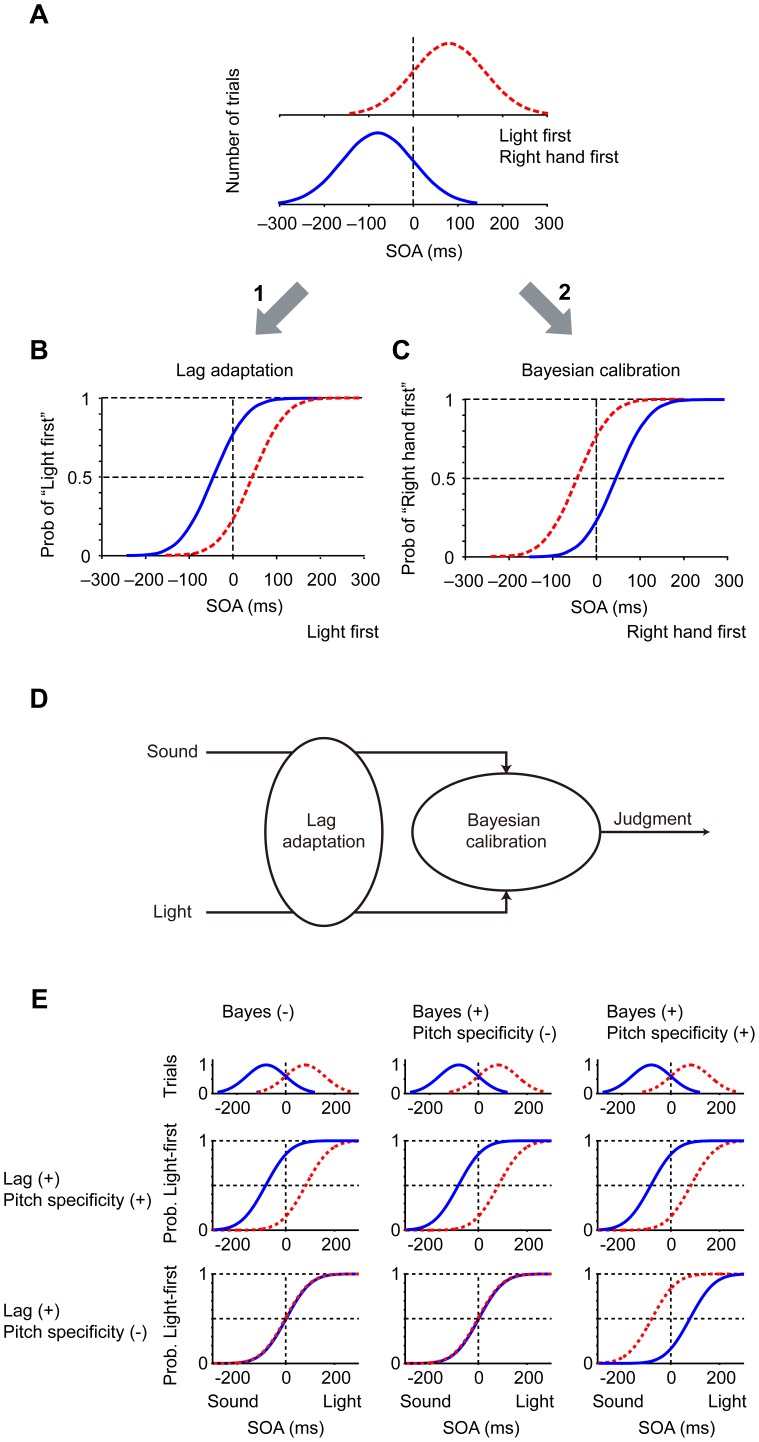
Two opposing calibrations of simultaneity in audiovisual (B) and tactile (C) temporal order judgments. (A) Examples of biased distribution of stimulation intervals: Gaussian distributions with positive (red dashed) and negative (blue solid) peaks. Positive interval shows “light first” in audiovisual, and “right hand first” in tactile temporal order judgments. (B,C) Opposing shifts of psychometric functions under the biased distributions in audiovisual (B) and tactile temporal order judgments (C). The probability of “light first” (B) and “right hand first” (C) judgments (ordinate) is plotted against the stimulation interval (abscissa; stimulus onset asynchronies, SOAs). Note that the point of simultaneity, as indicated by the intersection of a psychometric function with P  =  0.5, shifted toward the peak of each Gaussian distribution in audiovisual (B, lag adaptation) but away from the peak in tactile temporal order judgments (C, Bayesian calibration). (D) A serial model for lag adaptation and Bayesian calibration. The order of the two systems could be the other way around. A constant time lag between a sound-light pair is adjusted before (or after) the signals enter Bayesian calibration mechanism. (E) Predictions of shifts in the mixed condition (second experiment). Six predictions are shown in a two-by-three factorial manner according to whether lag adaptation was pitch-specific or not (rows), and whether Bayesian calibration existed, existed but was not pitch-specific, or was pitch-specific (columns). Note that the point of simultaneity is expected to move away from the Gaussian peak only when Bayesian calibration is working in a pitch-specific manner, but lag adaptation is not pitch-specific (second row, third column).

We previously reported these contrasting findings by using stimuli whose stimulation interval was sampled from a Gaussian distribution (Figure 1A) [9]. Let us assume, for example, that the stimulation interval of audiovisual stimuli was sampled from a Gaussian distribution with a positive peak such that light preceded sound by 80 ms on average (red dashed curve in Figure 1A). We found that the point of simultaneity, as indicated by the intersection of a psychometric function with P  = 0.5, shifted toward the peak of the Gaussian (arrow 1, red dashed curve in Figure 1B) so that the probability of “light-first” answers *decreases*. The results agreed well with the prediction of lag adaptation in that stimuli with the most frequent order tended to be perceived as simultaneous.

On the other hand, when the interval of tactile stimuli was sampled from the same Gaussian distribution, that is, the right hand stimulus preceded left hand stimulus by 80 ms on average (red dashed curve in Figure 1A), the point of simultaneity shifted away from the peak in the opposite direction (arrow 2, red dashed curve in Figure 1C; mostly right-hand-first). The opposite perceptual changes conformed to predictions derived from Bayesian integration theory [10], [11], [12], in that participants tended to perceive simultaneous stimuli as right hand first, that is, as the most frequent order. In other words, the prior probability of “right-hand-first” stimuli increases so that the probability of “right-hand-first” answers *increases*. We called this Bayesian calibration [9]. These contrasting findings raised a critical question as to whether and how these counteracting mechanisms of calibration, lag adaptation and Bayesian calibration are enacted in the brain.

Recent studies have examined the calibration of simultaneity in response to other combinations of sensory modalities, and have produced contradictory results. Some studies [2], [13], [14] reported lag adaptation in regard to sound and touch, but other studies did not [3], [15], [16]. Likewise, some studies found lag adaptation in response to light and touch [2], [17], whereas others did not [3], [18]. These findings may seem confusing, but they become more reasonable upon acknowledgment that both mechanisms operate in the brain.

Lag adaptation is considered to be useful to unify asynchronous sound and light that actually originated from a single event. On the other hand, Bayesian calibration is useful when sound and light have originated from two different events. We previously proposed a serial model that combined lag adaptation and Bayesian calibration (Figure 1D), which takes both occasions into account. When two signals are unified by the “lag adaptation” unit, the Bayesian calibration system perceives the temporal difference as zero, so there would be no effect of Bayesian calibration. On the other hand, when it is obvious that the sound and light came from different sources, there would be no lag adaptation to unify them and Bayesian integration emerges itself. We also showed, in theory, that the size and direction of shift in the point of simultaneity ranges from full lag adaptation to full Bayesian calibration, depending on the strength of lag adaptation (for mathematical detail, see Supplementary methods in [9]). This led us to hypothesize that Bayesian calibration is at work even during audiovisual temporal order judgments, but that the effect is concealed behind lag adaptation [9].

To test this hypothesis, we devised a method that would cancel lag adaptation while leaving Bayesian calibration intact. We used two pitches of sound for this purpose (cf. [19], [20]) and associated one (e.g. high tone) with a light-first Gaussian distribution (red dashed Gaussian in Figure 1E) and the other (e.g. low tone) with a sound-first Gaussian distribution (blue Gaussian). If lag adaptation discriminates between the two tones (Lag (+), pitch specificity (+); first row in Figure 1E), full lag adaptation is expected to occur for each sound and Bayesian calibration would be left concealed behind lag adaptation. In this case, we would not be able to show that Bayesian calibration is at work.

However, multimodal neurons in the superior colliculus and insula, which have been implicated as candidates for lag adaptation [1], have no clear frequency tuning to pure tones [21], [22], [23]. We therefore expected that lag adaptation would be broadly tuned for pitch, and lag adaptation would be cancelled out (second row in Figure 1E). In this case, results depend on whether Bayesian calibration mechanism is actually working and whether the mechanism is pitch specific. When lag adaptation is not pitch-specific but Bayesian calibration is operating in a pitch-specific manner (second row, third column), the point of simultaneity would be expected to move away from the Gaussian peak, and we are able to show that Bayesian calibration is at work. We tested the hypothesis and found that Bayesian calibration operated even during judgments regarding audiovisual temporal order that have been reported to show the most evident lag adaptation.

## Methods

### Participants

Thirty paid volunteers (19 men and 11 women) participated. All were naïve as to the purpose of the experiments, and were strongly right-handed according to the Edinburgh Inventory [24]. Approval of the study was granted by the internal review board of National Institute of Advanced Science and Technology (AIST), and all participants provided written informed consent in accordance with institutional guidelines.

### Apparatus and Task Procedures

Participants were seated in a semi-dark room and judged the order of a tone pip (1046 or 1480 Hz, 75–90 dB SPL, 10-ms duration with 2.5-ms ramps at both ends) delivered through headphones (Sennheiser HD650) and a visual stimulus from a red light-emitting diode (LED, 60 cm in front of the participant), while fixating on the LED. Participants responded in a forced choice manner within 3 s after the delivery of the second stimulus by pressing the right or the left button according to whether they judged as sound-first or light-first. Button assignment was counterbalanced among participants in each experiment. Participants rested more than 3 min every 100 trials.

### Sound- and Light-first Conditions (Experiment 1)

Eight paid volunteers (four men and four women) participated. Stimulus onset asynchronies (SOAs) were assigned from 11 intervals (−280, −240, −200, −160, −120, −80, −40, 0, 40, 80, and 120 ms) in the sound-first condition, and from 11 intervals (−120 to 280 ms) in the light-first condition. Positive intervals showed that the light onset was earlier than the sound onset. The 11 intervals used were each presented a different number of times to generate a Gaussian distribution of the probability of being exposed to a given lag. The Gaussian was centered on either −80 or +80 ms (sound-first or light-first conditions, respectively) and had a standard deviation (SD) of 80 ms. To create this, the intervals in the range were presented 2, 2, 6, 12, 18, 20, 18, 12, 6, 2, and 2 times in each 100-trial block, respectively. Four participants participated in four blocks in the sound-first condition, and then participated in another four blocks in the light-first condition; the procedure was reversed for the remaining four participants. Low- and high-tone pips (1046 and 1480 Hz) were presented in the sound- and light-first condition, respectively, for four participants and in the reverse for the remaining four participants. Combinations of the order of experiments and the assignment of pitch were counterbalanced among participants.

### Mixed Condition (Experiment 2)

Eight new paid volunteers (five men and three women) participated. Conditions were the same as those in the first experiment except that both low- (1046 Hz) and high-pitch (1480 Hz) audiovisual stimuli were presented in the same block in random order. Each 100 trial block consisted of 50 low- and 50 high-tone trials. The stimulation intervals for low-tone trials were sampled from one of the two biased distributions and those for high-tone trials were sampled from the other. All participants participated in 24 blocks of trials (2400 trials) over three days, at eight blocks per day.

### Mixed Condition with a Non-biased Distribution (Experiment 3)

To estimate a baseline shift in the point of simultaneity without lag adaptation or Bayesian calibration, we added another experiment that was similar to Experiment 2 but differed in that SOAs for low-tone trials and those for high-tone trials were both sampled from a distribution with a zero mean. Eight new paid volunteers (six men and two women) participated. Each 100 trial-block consisted of 50 low- and 50 high-tone trials in random order, as in the second experiment, but stimulation intervals were sampled from a single distribution with a zero mean that was generated by adding the two biased Gaussian distributions. SOAs were assigned from 15 intervals (−280 to +280 ms).

### Mixed Condition with Adaptation/test Subdivisions (Experiment 4)

Six new paid volunteers (four men and two women) participated. Six to ten adaptation stimuli (6, 8 or 10), half of which were sound first (−235 ms, n  =  3, 4 or 5) and the other half were light first (+235 ms, n  =  3, 4 or 5), were presented before presenting a single test sound-light pair, so that adaptation periods were separated from tests. During adaptation, the high tone (1480 Hz) preceded a light (−235 ms) and the low tone (1046 Hz) followed it (+235 ms) in three participants, and vice versa in the others. The SOAs of ±235 ms were adopted after Fujisaki et al. (2004) [1] that reported lag adaptation. Adaptation stimuli with high- and low-tone of the same number were randomly presented in each adaptation period. Each stimulus pair was presented every 1.2–1.8 s. Participants were instructed to attend both audio and visual stimuli as in Fujisaki et al. (2004). 0.4–0.7 s after the last adaptation stimulus pair was presented, two green LEDs on both side of the red LED (for visual stimulus) flashed for 50 ms to warn participants about the beginning of a test trial. A test stimulus pair was delivered 0.6–0.9 s later. In each test trial, one stimulus pair was chosen from 16 different pairs of 2 pitches (high or low) by 8 SOAs (−280, −200, −120, −40, +40, +120, +200, +280 ms). It is worth noting that SOAs were balanced so as not to induce any response biases. Participants were required to judge the order of two stimuli in a forced choice manner after the delivery of the second stimulus. The 16 pairs were presented once for each block, and each participant participated in 12 blocks (12 × 16 = 192 test trials).

### Analysis

The response data were sorted according to stimulation interval in order to calculate order-judgment probabilities that the light was earlier than the sound in each of the high and low-tone trials. We fitted the order-judgment probabilities by a cumulative density function of a Gaussian distribution:
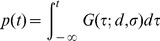
(1)where



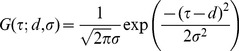
(2)In these equations, *τ, d*, and *σ* denote the stimulation interval, size of the horizontal transition, and temporal resolution, respectively. Matlab (optimization tool box) was used for fitting by adjusting *d*, and *σ* so as to maximize the log-likelihood.

## Results

### Sound- and Light-first Conditions (Experiment 1)

**Figure 2:**
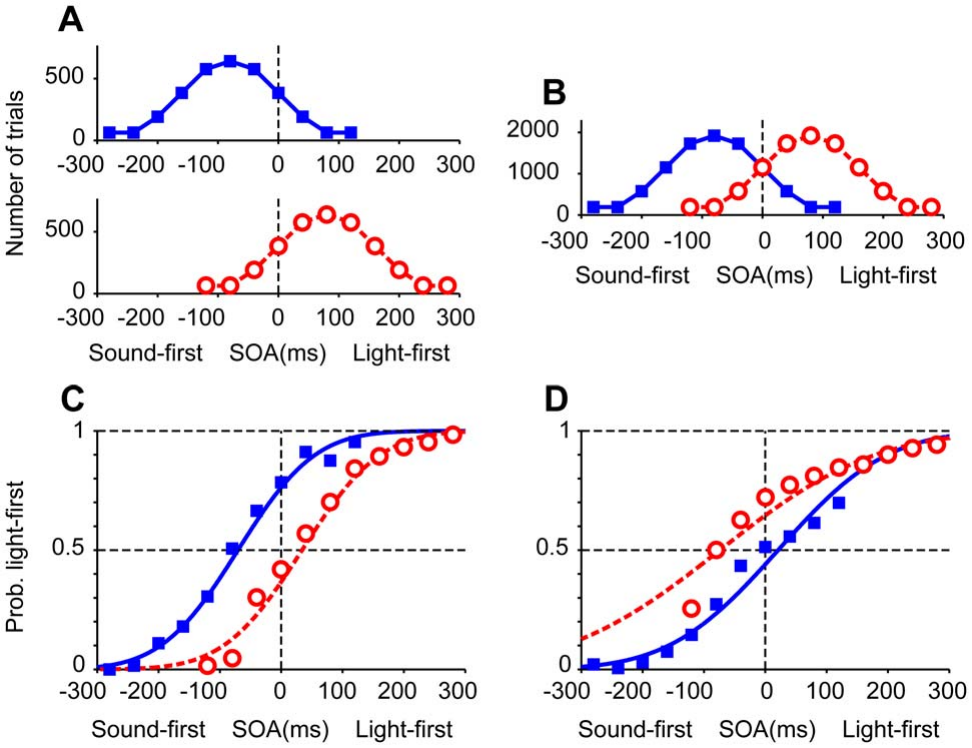
Two opposing calibrations of simultaneity in audiovisual temporal order judgments. (A, B) SOAs between a light stimulus and a tone pip (1046 or 1480 Hz) were sampled from one of two Gaussian distributions, one biased toward sound-first intervals (mean, −80 ms; squares and blue solid curves) and the other toward light-first intervals (mean, +80 ms; open circles and red dashed curves). The two tones were associated with different distributions (sound-first or light-first), and were alternated in blocks of 100 trials in the first experiment (A), but intermingled in the second experiment (B, mixed condition). (C) Shifts in the point of simultaneity in the first experiment, favoring lag adaptation. Each symbol represents 64–640 judgments from eight participants, totaling 6,400 trials. Trial numbers for each symbol are shown in (A). (D) Shifts in the point of simultaneity in the mixed condition (Experiment 2), favoring Bayesian calibration. Each symbol represents 192–1,920 judgments from eight participants, totaling 19,200 trials. Blue and red curves in C and D show the results of model fitting for the pooled data from all participants (maximum likelihood estimation).

**Figure 3:**
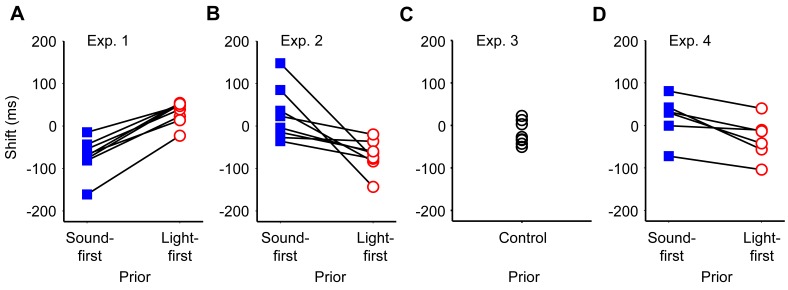
Shifts in the point of simultaneity calculated for each individual participants. Each panel shows data from Experiment 1 (A), 2 (B), 3 (C), and 4 (D). Note that all participants yielded data in agreement with lag adaptation in Experiment 1 (A), but all participants yielded those in agreement with Bayesian calibration in Experiment 2 (B) and 4 (D).

In the first experiment, participants judged the order of a pair of sound and light stimuli under two different conditions. In the sound-first condition, stimulation intervals were selected from a Gaussian distribution biased toward “sound first” (blue solid Gaussian curve in Figure 2A; mean ± SD, –80±80 ms); and in the light-first condition, stimulation intervals were selected from a Gaussian distribution biased toward “light first” stimuli (red dashed Gaussian curve in Figure 2A; +80±80 ms). Each prior distribution was associated with one of two tone pitches (1046 or 1480 Hz), such that the participant heard a single pitch in each block of trials (n  =  100). Response curves in the two conditions were separated from each other in the direction as predicted from lag adaptation (Figure 2C). The point of simultaneity in the sound-first condition was nearer to the peak of the sound-first Gaussian (blue solid curve in Figure 2C; *d*  =  –70 ms, *σ*  =  99 ms) and the point of simultaneity in the light-first condition was nearer to the peak of the light-first Gaussian (red dashed curve in Figure 2C; *d*  =  +34 ms, *σ*  =  100 ms), when the data were pooled for all participants. When the data were analyzed participant by participant, the point of simultaneity calculated for each participant (n  =  8; Figure 3A) was significantly smaller in the sound-first condition (mean ± SEM, –71±15 ms) than in the light-first condition (+32±9 ms; *P*  =  0.0000096, paired *t*-test). The results confirmed that lag adaptation occurred in audiovisual temporal order judgments such that the frequent lag was ignored. It is worth noting, however, that the points of simultaneity in the two conditions were not symmetric but biased toward the sound-first (negative) direction, as a whole. This finding is consistent with those reported in previous studies [1], [2], [3], [4], [5], [6], [7], [8] (but see [25]).

### Mixed Condition (Experiment 2)

In the second experiment (mixed condition, Figure 2B), stimulation intervals were sampled randomly from either of the two Gaussian distributions that were again associated with low and high tones. For example, a low tone was generally preceded by a light (mean interval  =  +80 ms), a light was generally preceded by a high tone (mean interval  =  –80 ms), but low-tone trials were intermingled with high-tone trials such that the mean interval equaled zero. Figure 2D shows response curves generated separately from the data in the sound-first (blue solid line, tone 1) and light-first (red dashed line, tone 2) trials. Response curves in the two conditions were separated in the direction as predicted from Bayesian calibration, such that the relative positions of the two curves were reversed in comparison to those in the first experiment (compare Figures 2C and D). The point of simultaneity in the sound-first trials (+21 ms, blue solid curve in Figure 2D), was greater than that in the light-first trials (–74 ms) in Experiment 2 (red dashed curve in Figure 2D. When the data were analyzed participant by participant, the points of simultaneity calculated for each participant (n  =  8; Figure 3B) were significantly greater in the sound-first condition (mean ± SEM, +26±22 ms) than in the light-first condition (–70±13 ms; *P*  =  0.0157, paired *t*-test). It is noteworthy that the reverse occurred in the second experiment merely by mixing sound-first and light-first trials, which had been separated into different blocks in the first experiment.

### Mixed Condition with a Non-biased Distribution (Experiment 3)

**Figure 4:**
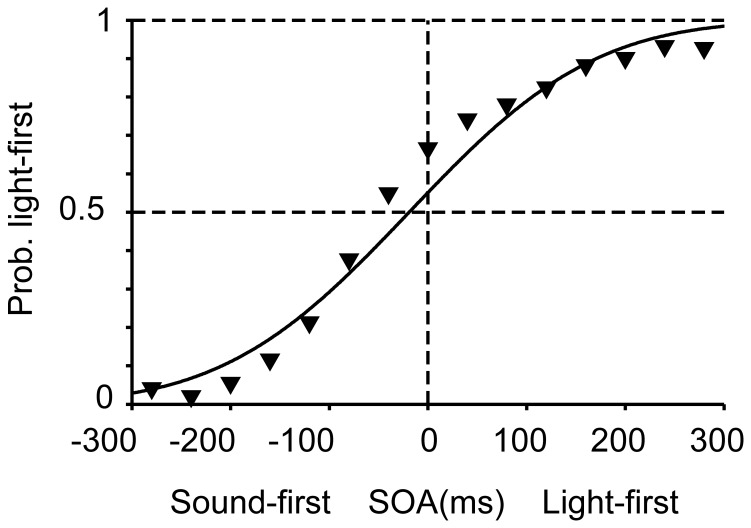
Audiovisual temporal order judgments without bias of stimulation intervals (Experiment 3). Each symbol represents 192–2,304 judgments from eight participants, totaling 19,200 trials. A black curve shows the result of model fitting for the pooled data from all participants.

In the third experiment, the pitch of the auditory stimulus was randomly determined, whereas all other conditions were identical to the second experiment. The point of simultaneity in this condition, calculated for the pooled data from all participants, was –19 ms (Figure 4; *σ*  =  148 ms) and fell between the two sigmoid curves observed in the mixed condition (Figure 2D). When the data were analyzed participant by participant, the points of simultaneity distributed from –51 ms to +22 ms (n  =  8; Figure 3C), and the mean (−19 ms) was not significantly different from zero (SEM  =  10 ms).

### Mixed Condition with Adaptation/test Subdivisions (Experiment 4)

**Figure 5:**
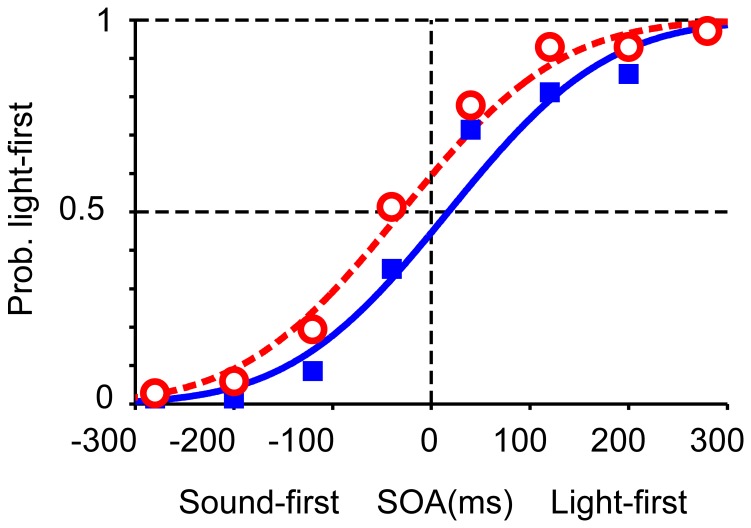
Shifts in the point of simultaneity in the Experiment 4, again favoring Bayesian calibration. In the experiment, each test stimuli was delivered after 6–10 adaptation stimuli, SOAs of which were sampled from −235 ms or +235 ms. Each symbol represents 72 judgments from six participants. Curves show the results of model fitting for the pooled data from all participants.

In the fourth experiment, we separated test trials from adaptation stimuli. Six to ten adaptation stimuli included both sound-first (−235 ms) and light-first (+235 ms) stimuli, each of which was associated with the high- or low-pitch sounds. In the test trials, SOAs were balanced so as not to induce any response biases. The arrangement of the two points of simultaneity was again opposite to that expected from lag adaptation. The point of simultaneity in test trials with the tone associated with the sound-first stimulus (−235 ms) was +17 ms (blue solid line, Figure 5; *σ*  =  127 ms), and that with the light-first stimulus (+235 ms) was −30 ms (red dashed line; *σ*  =  127 ms). The difference was +47 ms between the −235 ms and +235 ms conditions for the pooled data. When the data were analyzed participant by participant, the shifts calculated for each participant (n  =  6; Figure 3D) were significantly different accordingly (+19±18 ms vs. −31±17 ms; *P*  =  0.0112, paired *t*-test). It is worth noting that Fujisaki et al. (2004) [1] reported lag adaptation of similar sizes but in the opposite direction (−54-ms difference between the −235 ms and +235 ms conditions) when only one of the two adaptation stimuli (−235 ms or +235 ms) was used during adaptation. The results clearly show that the arrangement of the two points of simultaneity was inverted just by mixing sound-first and light-first stimuli, each of which was associated with either 1046 or 1480 Hz, during adaptation.

We should note that the effect size in Experiment 4 (+17 ms vs. –30 ms, a difference of 47 ms for the pooled data) was half as large as that in Experiment 2 (+21 ms vs. –74 ms, a difference of 95 ms). In Experiment 2, stimuli for tests were identical to those for adaptation. Accordingly, stimulations in test trials were biased in Experiment 2, but not in Experiment 4. It is possible that the bias in the stimulations in test trials in Experiment 2, which might have led to a kind of response bias, was a contributor to the larger effect size in Experiment 2 [9]. On the other hand, the effects observed in Experiment 4 cannot be explained by a simple “response bias”, because stimulations in test trials were not biased. In addition, the difference in the effect sizes may be explained in part by the difference in the distribution of SOAs. According to the serial Bayesian model, the larger the standard deviation of SOAs, the smaller the Bayesian shift becomes [9]. Because the standard deviation of SOAs in Experiment 4 (∼125 ms) was larger than that in Experiment 2 (∼80 ms), the model predicts that the Bayesian shift should be smaller in Experiment 4.

## Discussion

In the present study, we devised a new method for “canceling” lag adaptation using two pitches (1046 and 1480 Hz), which allowed us to successfully unmask Bayesian calibration operating behind lag adaptation. The results were basically unchanged whether we separated adaptation stimuli from test stimuli or not. It is worth noting that Bayesian calibration was observed even when the SOAs of test stimuli were balanced so as not to induce any response biases. The results exclude the possibility that the observed effects resulted from a simple “response bias”. Our results clearly show that both mechanisms were operating during audiovisual temporal order judgments, which have been reported to show the most evident lag adaptation, and that the final direction of calibration depended on the strength of the lag adaptation mechanism. In audiovisual adaptation, lag adaptation was sufficiently strong to conceal Bayesian calibration, but in tactile temporal order judgment lag adaptation was so small that it revealed Bayesian calibration. In other modalities, such as audio-tactile or visuo-tactile, the strength of lag adaptation may be intermediate, resulting in the previously observed variation [2], [3], [15], [16], [17], [18]. Thus, our study provides a resolution to the current debate regarding whether lag adaptation or Bayesian calibration is at work during temporal order judgment; our results demonstrate that both are in operation.

We previously showed, in theory, that the observed point of simultaneity (*d_u_*) is expressed as the weighted sum of the effect of Bayesian calibration (the first term) and that of lag adaptation (*c* in the second term) as follows:
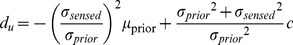
(3)


where *σ_sensed_*, *σ_prior_*, and *µ_prior_* denote the standard deviation of the sensed stimulation interval around the true interval, the standard deviation of the true stimulation interval, and the mean of the true stimulation interval, respectively (see Supplementary methods in Miyazaki et al., 2006 [9] for details). When the size of lag adaptation (*c*) moves from zero to *µ_prior_*, the point of simultaneity (*d_u_*) moves from full Bayesian calibration (first term) to full lag adaptation (*µ_prior_*). All but one of previous studies using a pair of sound and light, including the present study, reported lag adaptation. This indicates that lag adaptation in regard to a pair of sound and light is always strong enough to conceal Bayesian calibration. This is reasonable, because sound arrival could be delayed in our daily life by as large as 100 ms from the arrival of the light when the source of the sound and light is 30 m away from the observer. On the other hand, lag adaptation is less evident in other combinations of sound and touch, or touch and light. This is probably because there is smaller time lag in these combinations than audiovisual pairs.

Harrar and Harris (2005) used a single pair of sound/light stimuli and reported a shift in the point of simultaneity that conformed to Bayesian calibration [18]. However, the same authors reported a shift that conformed to lag adaptation in their later study [3]. The difference may be due to a difference in the way of presenting the sound and the light. In their later study that conformed to lag adaptation [3], the light and the sound was localized close-by in front of the participant so that they can be perceived to have originated from a single source. In their earlier study that conformed to Bayesian integration [18], in contrast, the light was mounted on a finger while the sound was delivered through headphones on the ears. The difference of localizations of the stimuli might have made the light and sound perceived as two distinct events, in which case lag adaptation was likely to be suppressed to reveal Bayesian adaptation. From the considerations, we predict that Bayesian calibration would be observed without using two pitches of sound when the sound source is separated far from the light source beyond the limit of spatial ventriloquism.

In addition to showing that both mechanisms are in operation in audiovisual temporal order judgments, our results show that Bayesian calibration, but not lag adaptation, is sensitive to pitch. In the second experiment, shifts in response curves generated separately for low- and high-tone trials (for light- and sound-first trials) were expected to differ according to whether lag adaptations were pitch-specific (Figure 1E). If lag adaptation discriminated between the two tones (first row in Figure 1E), lag adaptation would be observed for each tone such that the point of simultaneity shifted toward each peak, as was observed in the first experiment (Figure 2C). The results would be unchanged, irrespective of whether Bayesian calibration was at work (three columns in the first row), because full lag adaptation would have canceled Bayesian calibration, as explained above by using the equation (3).

On the other hand, if lag adaptation does not discriminate between two tones, lag adaptation should have disappeared because there would have been no bias in the mean interval as a whole. In this case (second row in Figure 1E), no shift in response curve would have been observed if there were no Bayesian calibration mechanism for the audiovisual stimulus pair (first column). Even if there were a Bayesian calibration, no shift would have occurred if the Bayesian calibration were not pitch-specific (second column). Only when Bayesian calibration was operating in a pitch-specific manner would the point of simultaneity be expected to move away from the Gaussian peak (third column). Our results are consistent with the schema shown in the last panel in Figure 1E (second row, third column), indicating that lag adaptation was not pitch-specific and that Bayesian calibration was actually operating in a pitch-specific manner.

It should be reminded, however, that our results are not sufficient to conclude that lag adaptation is completely insensitive to pitch even in the tone pair of 1046 Hz and 1480 Hz, to say nothing of other tone pairs with larger differences. Nevertheless, our results show that lag adaptation was less specific to pitch than Bayesian calibration, at least for the two pitches, so that lag adaptation was attenuated to the point where Bayesian calibration emerged. The same reservation applies to the pitch specificity in Bayesian calibration. Detailed pitch dependency should be examined in the future.

Results of two recent studies are relevant to pitch specificity in audio-visual lag adaptation or Bayesian integration. Heron et al. (2012) [4] used a low frequency pair (500 Hz - low spatial frequency) and a high frequency pair (2000 Hz - high spatial frequency), and reported no significant changes in the point of simultaneity. According to our serial model (Figures 1D and E), the result may suggest that there was no pitch specificity in either of lag adaptation or Bayesian calibration. We suggest another possibility that lag adaptation was mildly pitch specific so that lag adaptation and pitch specific Bayesian calibration cancelled out each other. Assuming the latter possibility, the difference between our results (lack of pitch specificity in lag adaptation) and their results (mild pitch specificity in lag adaptation if any), might be explained by the difference in the visual properties. In theirs, two audio-visual pairs were different not only in their pitches but also in their visual properties. So each pair of stimuli might be perceived as a single event, which might enhance lag adaptation. In ours, the light stimulus was shared among the two sound-light pairs. So the light stimulus cannot be associated with a low pitch sound, or high pitch sound, which might have inhibited lag adaptation.

Roseboom and Arnold (2011) [7] used a “male voice” - “male face movie” pair and a “female voice” - “female face movie” pair, and observed a significant difference in the point of simultaneity in the direction predicted from lag adaptation. The results may suggest that lag adaptation could be pitch specific. However, in their study, two pairs of stimuli were presented in different positions on the screen. So the results can be explained without assuming any pitch specificity but by just assuming that participants are able to adapt to different lags when audio-visual pairs are presented at different spatial positions. In fact, Heron et al. (2012) [4] reported in another experiment that participants were able to adapt to two different lags, when audio-visual stimuli, with a fixed frequency for each property, were presented at two different spatial positions with two different lags.

The recommended broad frequency tuning in lag adaptation would be advantageous for binding sound and light stimuli from any natural event occurring at some distance, because sound frequency cannot be foreseen before the event actually occurred. The signals should be bound together depending solely on the temporal difference and not on pitch. Bayesian estimation, on the other hand, improves our perception by making the most of previous experiences. Indeed, the learned prior distribution should be as detailed as possible to segregate multiple events. Thus, it is reasonable that the frequency tuning of Bayesian calibration would be finer than that of lag adaptation.

Subjective audiovisual simultaneity or temporal order may be represented by the pattern of activities over many neurons, each of which is sensitive to a certain range of temporal lags in audiovisual signals [1], [26]. Multimodal neurons in the superior colliculus and insula have been implicated as candidates underlying the perception of audiovisual simultaneity [27] and lag adaptation [1]. Neurons in these areas actually respond to combinations of sound and light stimuli, and their responses are modulated by the stimulation interval [28], [29], [30]. In general, auditory responses in these areas are broadly tuned. Some neurons in the superior colliculus are broadly tuned below 10 k Hz, as in cats [23], and have with bandwidths as large as six octaves, as in squirrel monkeys [21]. Many neurons in the insular cortex have no clear frequency tuning to pure tones, but are responsive to frequency-modulated sounds, as in squirrel monkeys [22]. The fact that lag adaptation does not discriminate between tones of 1046 and 1480 Hz may be consistent with the contribution of multimodal neurons in areas with broad frequency tuning.

On the other hand, Bayesian calibration discriminated between tones of 1046 and 1480 Hz. As such, it follows that the neural mechanisms underlying Bayesian calibration involve multisensory neurons with sharp frequency tuning. Possible candidates include those in the primary and secondary auditory fields, where audiovisual interactions already occur with stimulus intervals of 20–80 ms [31] and fine frequency tuning exists along with associative representational plasticity [32]. These possibilities require additional research.

Bayesian framework has been successful in explaining many phenomena, from visual perception [33], [34], [35], [36], [37] to sensorimotor learning [38], [39]. On the other hand, most perceptual adaptations, including the lag adaptation as one example, can be termed as anti-Bayesian [40], because the direction of perceptual change is opposite to that expected from Bayesian inference.

It is worth noting, however, that both “Bayesian” and “anti-Bayesian” biases may be explained in terms of the Bayesian theorem, a product of not only the prior distribution but also the likelihood function. According to Sato and Aihara [41], [42], the former can be regarded as the learning of the prior distribution whereas the latter as the learning of the likelihood function.

In either case, the key question why there are two contrasting mechanisms in the brain has not been explicitly addressed so far, because two groups of tasks have been mutually exclusive. Our present study has shown for the first time, to our knowledge, that both mechanisms can be at work in a single perceptual task. We have further provided a way to cancel “anti-Bayesian” adaptation while leaving “Bayesian” calibration at work. Our present study has thus opened a possibility to explore neural mechanisms underlying Bayesian calibration and anti-Bayesian adaptation, or the learning of the prior and that of the likelihood, without any confounding factors resulting from task difference.

## References

[1] Fujisaki W, Shimojo S, Kashino M, Nishida S (2004). Recalibration of audiovisual simultaneity.. Nat Neurosci.

[2] Hanson JV, Heron J, Whitaker D (2008). Recalibration of perceived time across sensory modalities.. Exp Brain Res.

[3] Harrar V, Harris LR (2008). The effect of exposure to asynchronous audio, visual, and tactile stimulus combinations on the perception of simultaneity.. Exp Brain Res.

[4] Heron J, Roach NW, Hanson JV, McGraw PV, Whitaker D (2012). Audiovisual time perception is spatially specific.. Exp Brain Res.

[5] Heron J, Whitaker D, McGraw PV, Horoshenkov KV (2007). Adaptation minimizes distance-related audiovisual delays.. J Vis 7: 5 1–8.

[6] Machulla TK, Di Luca M, Froehlich E, Ernst MO (2012). Multisensory simultaneity recalibration: storage of the aftereffect in the absence of counterevidence.. Exp Brain Res.

[7] Roseboom W, Arnold DH (2011). Twice upon a time: multiple concurrent temporal recalibrations of audiovisual speech.. Psychol Sci.

[8] Vroomen J, Keetels M, de Gelder B, Bertelson P (2004). Recalibration of temporal order perception by exposure to audio-visual asynchrony.. Brain Res Cogn Brain Res.

[9] Miyazaki M, Yamamoto S, Uchida S, Kitazawa S (2006). Bayesian calibration of simultaneity in tactile temporal order judgment.. Nat Neurosci.

[10] Kersten D, Mamassian P, Yuille A (2004). Object perception as Bayesian inference.. Annu Rev Psychol.

[11] Knill DC, Pouget A (2004). The Bayesian brain: the role of uncertainty in neural coding and computation.. Trends Neurosci.

[12] Körding KP, Wolpert DM (2006). Bayesian decision theory in sensorimotor control.. Trends Cogn Sci.

[13] Keetels M, Vroomen J (2008). Temporal recalibration to tactile-visual asynchronous stimuli.. Neurosci Lett.

[14] Keetels M, Vroomen J (2008). Tactile-visual temporal ventriloquism: no effect of spatial disparity.. Percept Psychophys.

[15] Navarra J, Soto-Faraco S, Spence C (2007). Adaptation to audiotactile asynchrony.. Neurosci Lett.

[16] Stetson C, Cui X, Montague PR, Eagleman DM (2006). Motor-sensory recalibration leads to an illusory reversal of action and sensation.. Neuron.

[17] Takahashi K, Saiki J, Watanabe K (2008). Realignment of temporal simultaneity between vision and touch.. Neuroreport.

[18] Harrar V, Harris LR (2005). Simultaneity constancy: detecting events with touch and vision.. Exp Brain Res.

[19] Walker JT, Irion AL (1979). Two new contingent aftereffects: perceived auditory duration contingent on pitch and on temporal order.. Percept Psychophys.

[20] Walker JT, Irion AL, Gordon DG (1981). Simple and contingent aftereffects of perceived duration in vision and audition.. Percept Psychophys.

[21] Allon N, Wollberg Z (1978). Responses of cells in the superior colliculus of the squirrel monkey to auditory stimuli.. Brain Res.

[22] Bieser A (1998). Processing of twitter-call fundamental frequencies in insula and auditory cortex of squirrel monkeys.. Exp Brain Res.

[23] Hirsch JA, Chan JC, Yin TC (1985). Responses of neurons in the cat’s superior colliculus to acoustic stimuli. I. Monaural and binaural response properties.. J Neurophysiol.

[24] Oldfield RC (1971). The assessment and analysis of handedness: the Edinburgh inventory.. Neuropsychologia.

[25] Stone JV, Hunkin NM, Porrill J, Wood R, Keeler V (2001). When is now? Perception of simultaneity.. Proc Biol Sci.

[26] Roach NW, Heron J, Whitaker D, McGraw PV (2011). Asynchrony adaptation reveals neural population code for audio-visual timing.. Proc Biol Sci.

[27] Bushara KO, Grafman J, Hallett M (2001). Neural correlates of auditory-visual stimulus onset asynchrony detection.. J Neurosci.

[28] Benevento LA, Fallon J, Davis BJ, Rezak M (1977). Auditory-visual interaction in single cells in the cortex of the superior temporal sulcus and the orbital frontal cortex of the macaque monkey.. Exp Neurol.

[29] Loe PR, Benevento LA (1969). Auditory-visual interaction in single units in the orbito-insular cortex of the cat.. Electroencephalogr Clin Neurophysiol.

[30] Meredith MA, Nemitz JW, Stein BE (1987). Determinants of multisensory integration in superior colliculus neurons. I. Temporal factors.. J Neurosci.

[31] Kayser C, Petkov CI, Logothetis NK (2008). Visual Modulation of Neurons in Auditory Cortex.. Cereb Cortex.

[32] Weinberger NM (2004). Specific long-term memory traces in primary auditory cortex.. Nat Rev Neurosci.

[33] Adams WJ, Graf EW, Ernst MO (2004). Experience can change the ‘light-from-above’ prior.. Nat Neurosci.

[34] Guo K, Nevado A, Robertson RG, Pulgarin M, Thiele A (2004). Effects on orientation perception of manipulating the spatio-temporal prior probability of stimuli.. Vision Res.

[35] Kersten D, Mamassian P, Knill DC (1997). Moving cast shadows induce apparent motion in depth.. Perception.

[36] Rao RP (1999). An optimal estimation approach to visual perception and learning.. Vision Res.

[37] Weiss Y, Simoncelli EP, Adelson EH (2002). Motion illusions as optimal percepts.. Nat Neurosci.

[38] Körding KP, Wolpert DM (2004). Bayesian integration in sensorimotor learning.. Nature.

[39] Miyazaki M, Nozaki D, Nakajima Y (2005). Testing Bayesian models of human coincidence timing.. J Neurophysiol.

[40] Brayanov JB, Smith MA (2010). Bayesian and “Anti-Bayesian” Biases in Sensory Integration for Action and Perception in the Size-Weight Illusion.. J Neurophysiol.

[41] Sato Y, Aihara K (2009). Integrative Bayesian model on two opposite types of sensory adaptation Artificial Life and Robotics.

[42] Sato Y, Aihara K (2011). A bayesian model of sensory adaptation.. PLoS One.

